# Root caries analysis in working population of 35-44 years of age (Spain)

**DOI:** 10.4317/medoral.21685

**Published:** 2017-08-16

**Authors:** Cristina Saura-Moreno, Maria-Victoria Cortés-Arcas, Ana Fernández-Meseguer, Eva Calvo-Bonacho, Juan-Carlos Llodra-Calvo

**Affiliations:** 1 Department of Preventive and Community Dentistry. University of Granada. Faculty of Odontology. Campus de Cartuja PC18071. Granada. Spain; 2 Cualtis (previously named Sociedad de Prevención de Ibermutuamur), Madrid, Spain; 3 Ibermutuamur (Mutua Colaboradora con la Seguridad Social, nº 274), Madrid, Spain

## Abstract

**Background:**

The aim of this study was to analyse the influence of socio-demographic variables, toothbrushing frequency, frequency of snacking between meals, and tobacco and alcohol consumption, in root caries in the Spanish working population of Valencia and Murcia regions.

**Material and Methods:**

Cross sectional study of 458 workers 35-44 years of age, who underwent a routine work-related check-up, from June 2009 to April 2010, and were also examined, following the WHO methodology, by a calibrated dentist. Stratified random sampling. Participants fulfilled a questionnaire comprising demographic data, toothbrushing frequency, snacking frequency and tobacco and alcohol consumption.

**Results:**

The DFS index (root caries) in the employed population of 35-44 years was 0.45 ± 1.3, with a root caries prevalence of 18.6% and an active root caries prevalence of 13.5%. Higher root caries prevalence and active root caries prevalence were associated with male gender, manual occupations, foreign country of origin, lower levels of education and income, lower brushing frequency and higher frequency of snacking between meals. The DFS index was associated with all studied socio-demographic variables, but gender, and it was also associated with brushing frequency. The mean number of root decayed teeth was associated with all socio-demographic variables, but country of origin, and it was also associated with brushing frequency.

**Conclusions:**

Adult workers 35-44 years of age showed worse root condition in regard to caries than general population of this age cohort. In this study, the frequency of toothbrushing and snacking between meals were the variables that influenced more in root caries.

** Key words:**Root caries, working population, epidemiological studies, toothbrushing, snacking.

## Introduction

Increased life expectancy and higher percentage of natural present teeth in adulthood have led to greater prevalence of root caries, nowadays (1). These facts in conjunction with the multifactorial nature of root cares, and the complexity of its restoring treatment when subgingival lesion, justify the importance of studying root caries to prevent it.

At a national level, there have been published in Spain four studies on root caries in young adults (35-44 years of age) and senior adults (65-74 years) financed by the Spanish Dental Association (Consejo General de Colegios de Dentistas de España) and published in their own scientific magazine, Revista del Ilustre Consejo General de Colegios de Odontólogos y Estomatólogos de España (RCOE), in years 2000, 2005, 2010 and 2015. All of them followed the World Health Organization (WHO) methodology for Oral Health Surveys to perform pathfinder epidemiological surveys, thus, allowing us to compare their findings. At a regional level, in the East coast of Spain, there has only been one study published, conducted on the Autonomous Region of Valencia in 2006, on general population of 35-44 years of age (2).

Published literature on root caries is more numerous on the senior adults cohort, prevalence being in this age cohort higher (3,4). It might be noted, however, that one study tested the hypothesis that factors related with caries experience were different in mid-dle-aged adults from senior adults (5).

To date, no study has been focused specifically on root caries in working population, whereas published information about variables associated with root caries in young adults has been limited.

The objective of this study was to analyse the influence of socio-demographic variables, toothbrushing frequency, frequency of snacking between meals, and tobacco and alcohol consumption, in root caries in working population of 35-44 years of age.

## Material and Methods

- Study design

This cross sectional study was part of the WORALTH (Workers’ Oral Health) study. The methodology of the WORALTH study has been described elsewhere (6). Briefly, WORALTH was an oral epidemiological survey conducted on a representative sample of the Spanish employed population, in four major geographical areas with five age strata, from April 2008 to June 2011. A stratified sampling method was followed. Strata sample size was defined in proportion with the Spanish labour Force Survey, 2nd quarter (Instituto Nacional de Estadística 2008) by age, gender and occupation, at each geographical area.

The study followed the WHO criteria for Oral Health Surveys published in 1997.The protocol was reviewed and approved by Ibermutuamur Ethics Committee. Signed informed consents were obtained from all participants before enrolment in the study, in accordance with principles of good clinical practice (ICH / ISO 14155) and the Helsinki Declaration (2008).

- Sampling and selection

A representative sample of 458 subjects of 35-44 years employed in Valencia and Murcia regions was defined. Workers were approached on occasion of their work-related annual check-up. The inclusion process followed this procedure: 1) when workers underwent their routine medical check-up, the computer program at the admission desk detected if the combination of variables (age, gender and occupation) met any of the strata criteria in that geographical area (the order of selection was determined by arrival to admission desk and data input); 2) workers were given detailed information with the consent forms and, then, were led to an oral examination room, filling a questionnaire on oral health before the clinical examinations were carried out by a trained examiner, with an assistant. The number of natural present teeth was not used as an inclusion criterion. This procedure was followed until the complete sample strata were recruited.

- Data collection

Data were collected at one sampling point in Valencia region, and one sampling point in Murcia region, from June 2009 to April 2010, with standardized conditions of light source, equipment, instruments and position of subjects who were examined.

- Human resources. Training and calibration

Clinical examinations at both sample points were carried by the same dentist, assisted by a clerk. Training and calibration sessions were carried by an experienced WHO expert, who acted as a benchmark examiner (Gold Standard). In order to obtain inter-examiner agreement with the Gold Standard, Kappa index was measured, obtaining a 0.73 index, which is considered as “substantial agreement”, according to Landis & Koch’s scale (7). Intra-examiner variability was also measured throughout the data recruitment.

- Sociodemographic and health variables

Socio-demographic and health variables were obtained from the medical check-up and the oral health questionnaire: Country of origin (Spain; other country); Education level (Primary school, Secondary School, University); Net income level of the family unit (≤1,200, 1,200-3,600 and >3,600 euros/month); Employed adults were classified into two major categories according to the Spanish National Classification of Occupations (Instituto Nacional de Estadística 1994), white collar (non-manual occupations) and blue-collar (manual occupations); Toothbrushing frequency (More than once per day, Once per day, Less than once per day/do not brush); Frequency of eating/snacking between meals including any drink with the exception of water/tea/coffee without sugar (never or almost never, 1-2 times per day, 3-4 times/day, 5-6 times/day, >6 times/day); Tobacco consumption (never smoker, former smoker who quit at least 12 months ago, current smoker of ≤10 cigarettes per day, and current smoker of >10 cigarettes per day); Alcohol consumption (no consumption, occasional consumer, weekend consumer, daily consumer).

- Clinical variables

The criteria of the WHO (4th) edition were employed for diagnosis and coding. The tooth roots were examined. The indices calculated were: Decayed Filled Surface (DFS) (root caries), decayed (root), filled (root), root caries prevalence, active caries prevalence and mean number of natural present teeth excluding the wisdom teeth.

- Statistical analysis

Descriptive statistics were calculated for all variables. Percentages and 95% confidence intervals (95% CI) were used for categorical data, and means and standard deviations in the case of quantitative variables. The association of socio-demographic and health variables with root caries was tested by chi-squared test for categorical variables, and ANOVA for quantitative variables. Logistic regression analyses were performed with DFS (root)>0 as dependent variable; first, we calculated crude odds ratios (OR); secondly, an analysis entering all variables in the model; finally, a stepwise backward logistic regression was performed (WALD method) in order to reduce the number of covariables. A variable was removed from the model if its associated *p*-value was <0.10. All analyses were carried out using IBM SPSS Statistics 22.0.0 and Oracle database management system.

## Results

- Sample description

The socio-demographic characteristics of the sample, composed of 458 workers of 35-44 years of age were as follows: higher percentage of males than of females (61.6% vs. 38.4) as well as of manual occupations (60.5% manual vs. 39.5% non-manual). 82.1% of the workers were from Spain. The highest percentage of workers were included in the secondary studies (43.5%) and net family income of 1,201-3,600€ euros/month categories, followed by workers with primary

studies (31.0%) and net income ≤1200€/month.

- Root caries analysis

DFS index (root caries) in this sample of young adults was 0.45±1.3 (mean ± standard deviation), with 0.30±1 mean number of teeth with decay in the root, and 0.14±0.9 mean number of teeth with fillings in the root. 18.6% of workers showed, at least, one root carious lesion (whether active or treated) and 13.5% of workers showed active root caries (root caries>0). The mean number of natural present teeth, over a 28 maximum after excluding the wisdom teeth, was 25.8±3.3.

- Analysis of the association between socio-demographic variables and root caries

 Table 1 shows root caries findings according to gender, occupation, country of origin, level of studies and income. All variables showed statistically significant association (*p*<0.05) with both root caries prevalence, and active root caries prevalence, i.e., we observed higher prevalence in males, manual occupations, workers from other countries, primary studies and lower level of income. The mean number of teeth with decay in the root showed statistically significant association (*p*<0.05) with all variables but country of origin. We found association between DFS index (root caries) and all variables but gender.

**Table 1:**
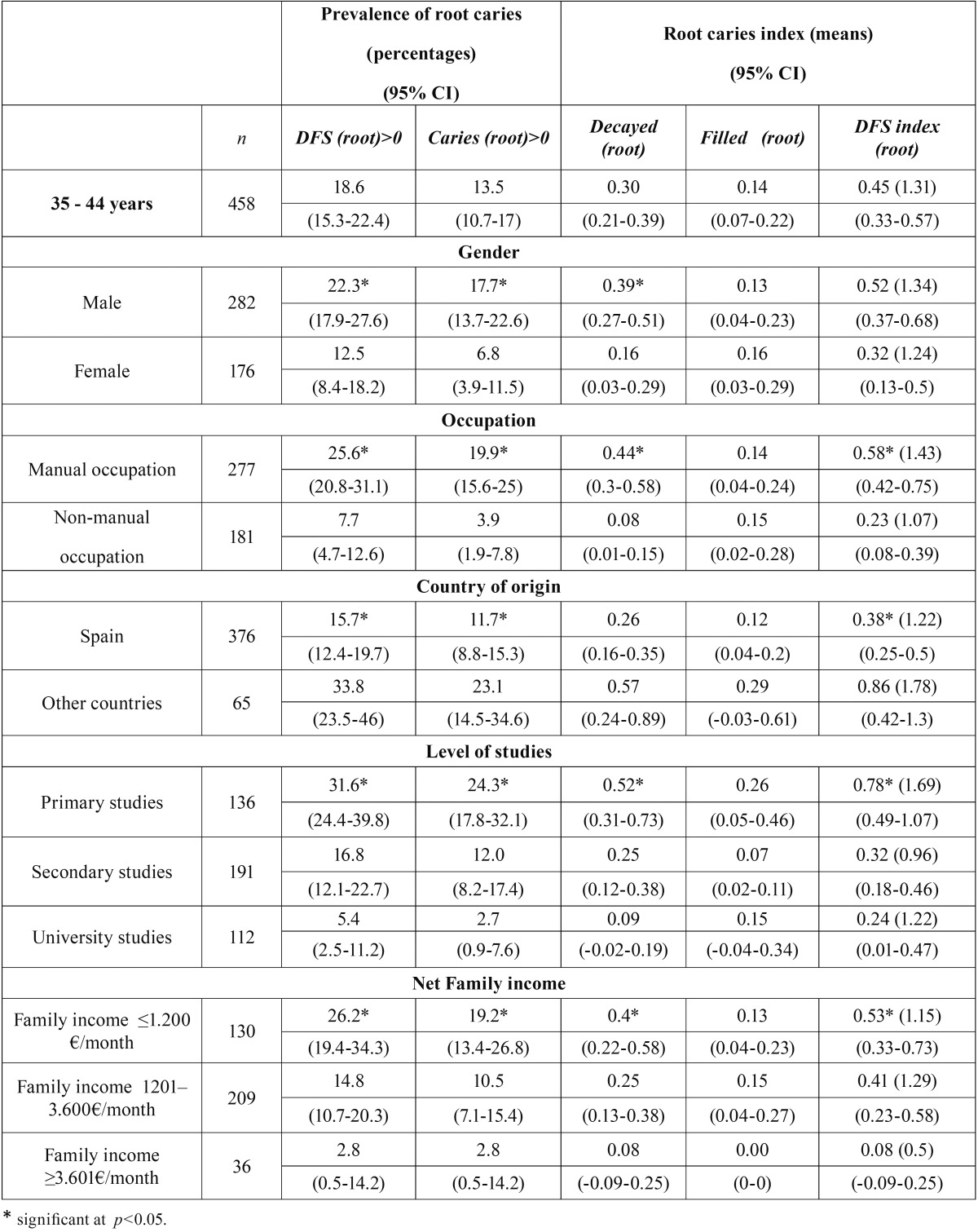
Root caries prevalence and index according to gender, occupation, country of origin, level of studies and net income level of the family unit.

- Analysis of the association between toothbrushing frequency and snacking between meals and root caries

 Table 2 shows root caries findings according to brushing frequency and frequency of snacking between meals (with the exception of water or coffee/tea without sugar). In this cohort, we found a statistically significant association (*p*<0.05) between brushing frequency and root caries prevalence, active root caries prevalence, mean number of teeth with decay in root and DFS index, i.e., we found better root health status in workers who brushed more than once per day, than those who only brushed once per day or those brushed less than once per day or did not brush.

**Table 2:**
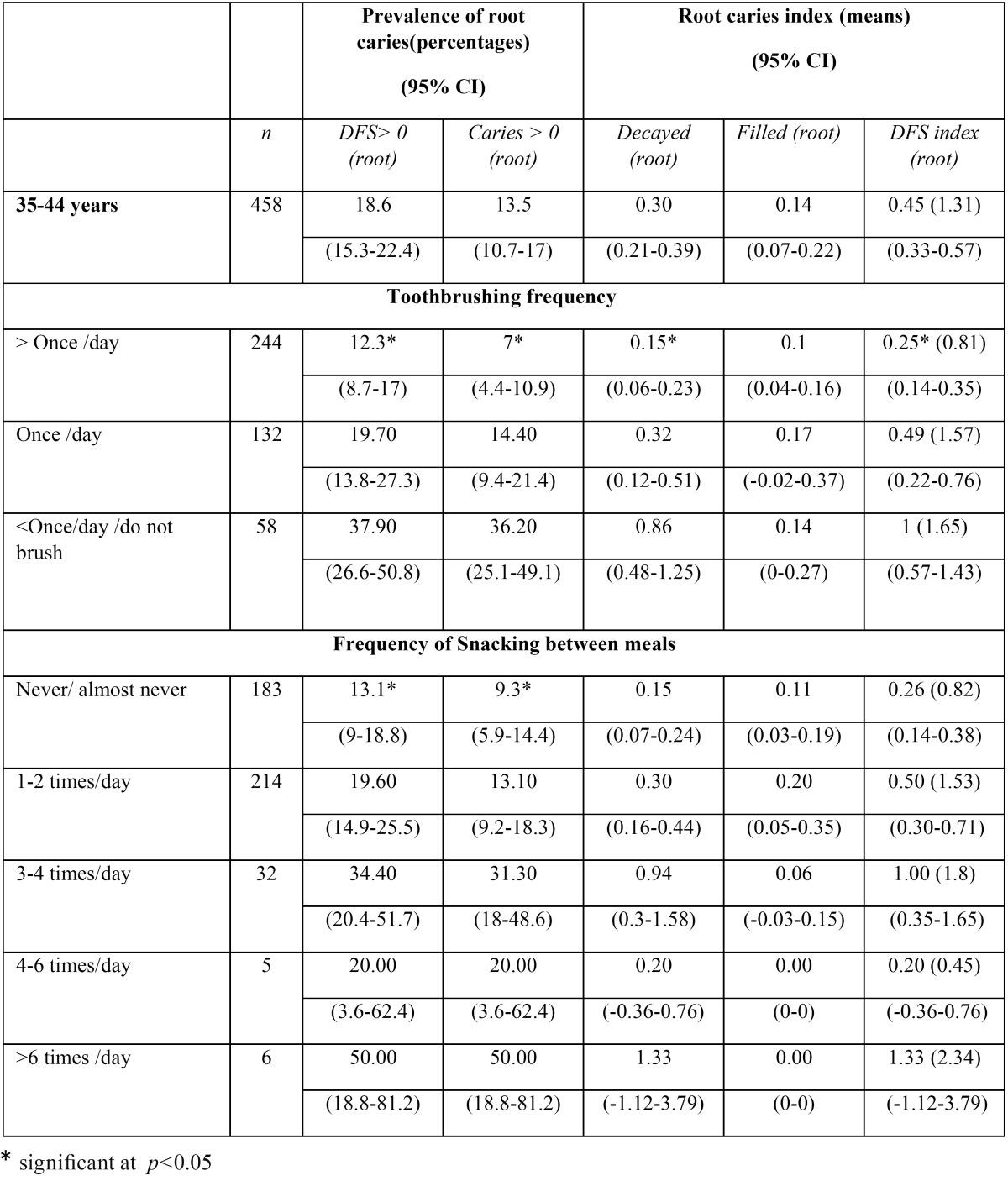
Root caries prevalence and index according to frequency of toothbrushing and frequency of eating/snacking between meals with the exception of water/tea/coffee without sugar.

We also found a statistically significant association (*p*<0.05) between root caries prevalence and active root caries prevalence with the frequency of snacking/eating between meals, i.e. subjects who “never or almost never” snacked between meals showed 13.1%, root caries prevalence and 9.3% active root caries prevalence versus those who snacked “more than 6 times per day”, who showed 50% of caries prevalence and root cares prevalence.

 - Analysis of the association between tobacco and alcohol consumption and root caries

 Table 3 shows root caries findings regarding tobacco and alcohol consumption. We only found a significant association (*p*<0.05) between tobacco consumption and active root caries prevalence, i.e., higher prevalence was found in current smokers of >10 cigarettes/day. Prevalence observed in heavy smokers was three times the one observed in former smokers and over two times the prevalence observed in light smokers and never smokers.

**Table 3:**
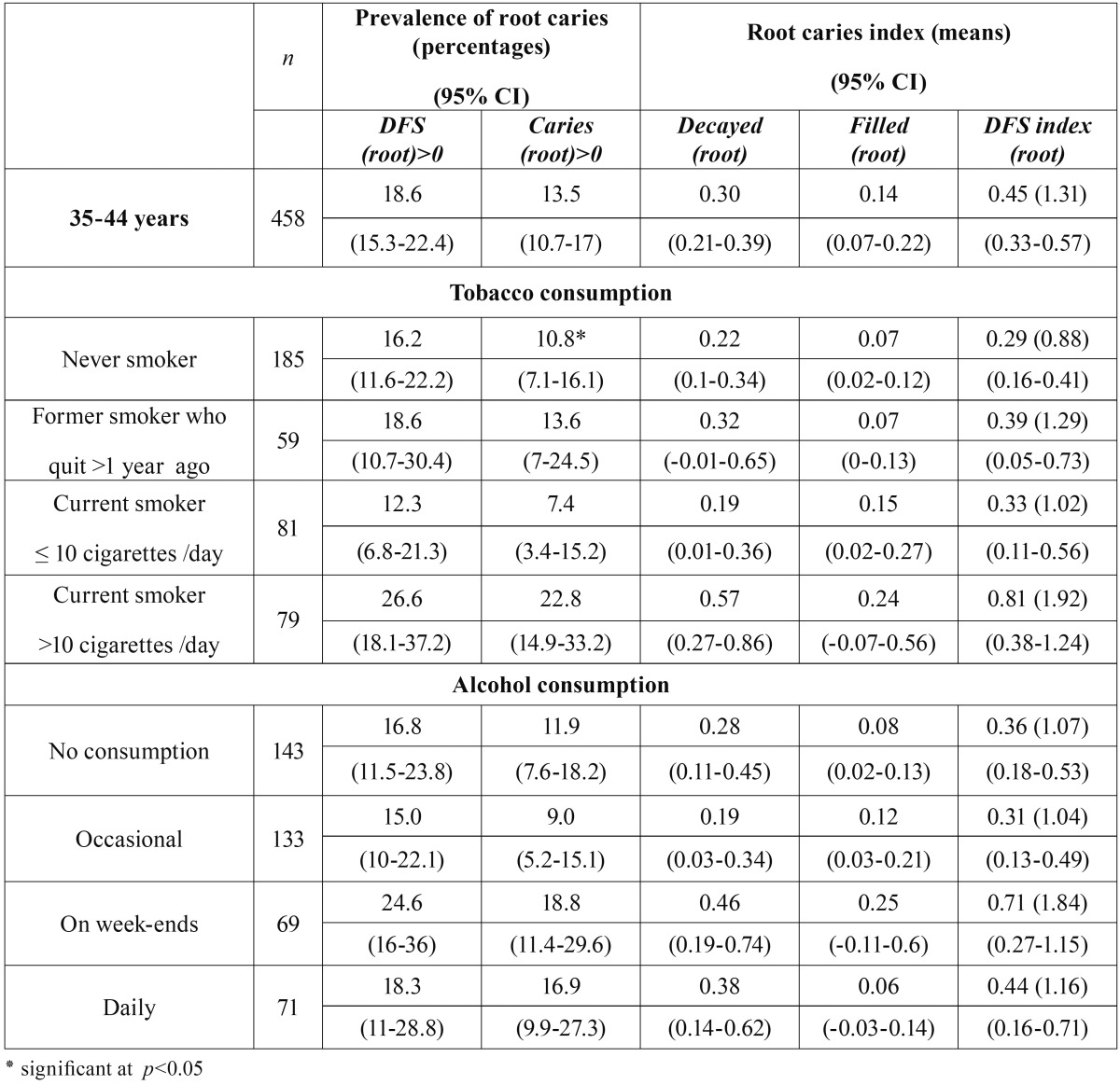
Root caries prevalence and index according to tobacco and alcohol consumption.

- Regression analysis

The logistic regression results for subjects with DFS (root) >0 are shown in  Table 4. We observed an association of all variables, but alcohol and tobacco consumption, if crudes OR were considered. The analysis including all variables lowered the effect of all them, with the exception of frequency of snacking between meals, which remained statistically significant. Finally, the stepwise backward model showed that workers who brushed less than once per day, compared with those who brushed more than once per day as reference category, had an increased probability of DFS (root) >0 [OR = 3.6 (1.5-8.4)]. The association of snacking/eating between meals ≥ 3 times per day and a higher prevalence of root caries was statistically significant, compared with those who never, or almost never did [OR = 3.6 (1.3-9.7)]; due to the small sample size of the last three categories for frequency of snacking between meals (with 32, 5 and 6 subjects, respectively) they were grouped in “≥ 3 times/day” in order to obtain robust results. Taking as reference workers with non-manual occupations, we found that manual workers had significantly increased probability of DFS (root) >0 [OR = 3.3 (1.4-7.8)]; it should be noted that the association with the country of origin showed a very low p-value, though.

**Table 4:**
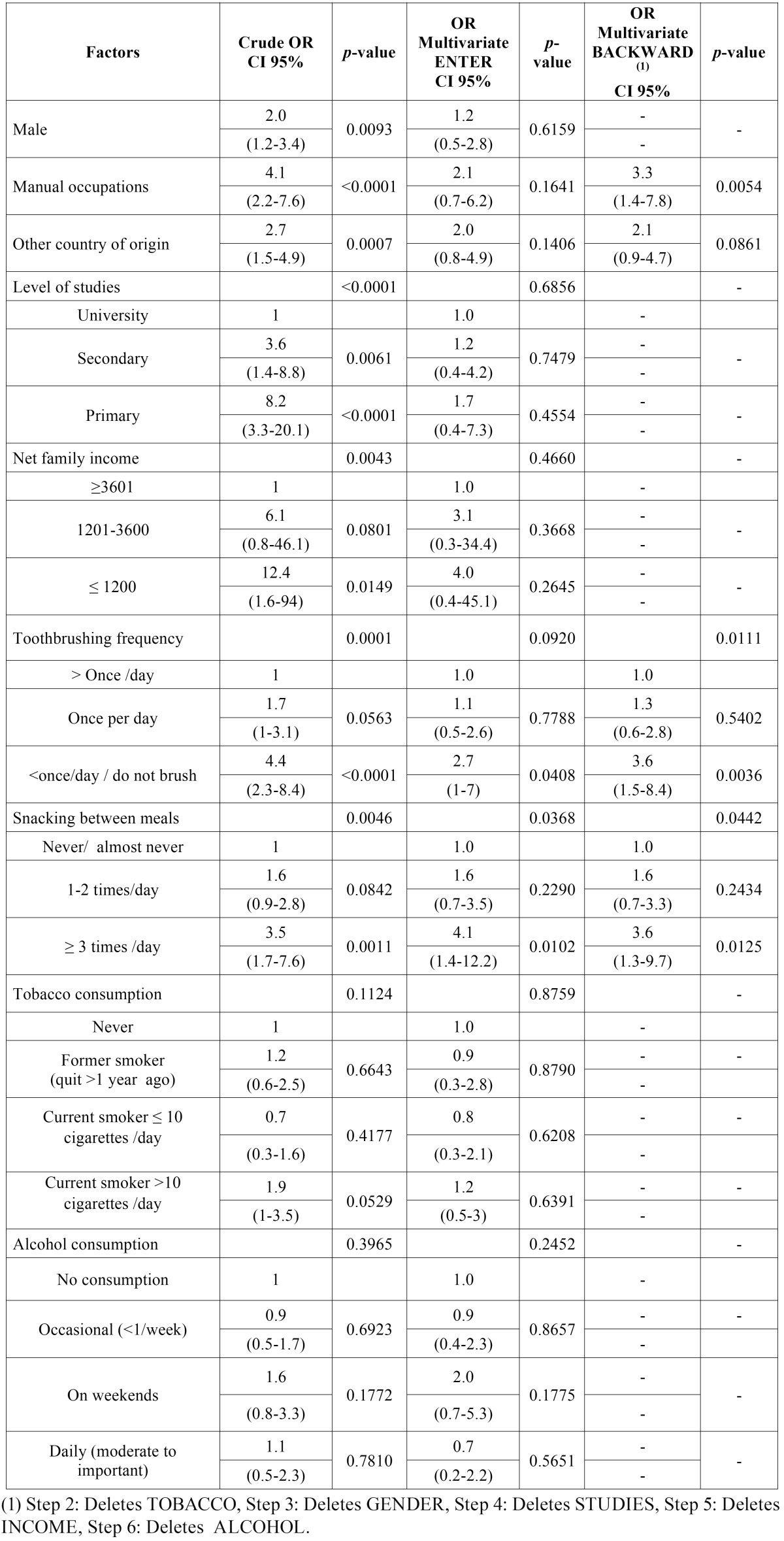
Logistic regression models of the effect of various factors on the odds of DFS (root) >0 expressed as odds ratio (OR) with 95% confidence interval (CI).

## Discussion

- Study limitations and methodological discussion

One strong points of this study emanates from using a self-administered questionnaire, as there is no potential bias on the way subjects answer it, increasing the perceived anonymity to truly answer it; this limits the potential risk of reacting to the environment, also referred as “the Hawthorne effect” (8), in which individuals tend to answer positively the positive habits and hide the negative ones. Moreover, as it was a close-ended questionnaire, with a set of response alternatives, it made it easier and simpler to use, to answer faster, and to process data from it, than if we would have used open-ended questions.

One limitation of this study could emanate from using the WHO standardized methodology for root caries diagnosis, which can underestimate the root caries disease, as it does not record its initial lesion, visible as a white spot. On the other hand, assessing caries prevalence can be affected by the presence of fillings of root, restored because of caries or other causes (9). Nevertheless, the methodology that was chosen has also brought some advantages like a fast way of identifying root carries lesions, reducing or eliminating potential risks of the oral exam, and, being a universal methodology, it has enabled us to analyze the description and evolution of root caries, comparing the results of this study with those of previous national studies in Spain, and with studies in other countries.

One of the limitations of DFS index, used to assess root caries, is that it equally ponders decayed and filled teeth. Nevertheless, its strong point is that it is universally used.

However, comparing this study with other studies should be made with caution, as working population is only a segment of general population.

- Comparison with national surveys data

**Figure 1:**
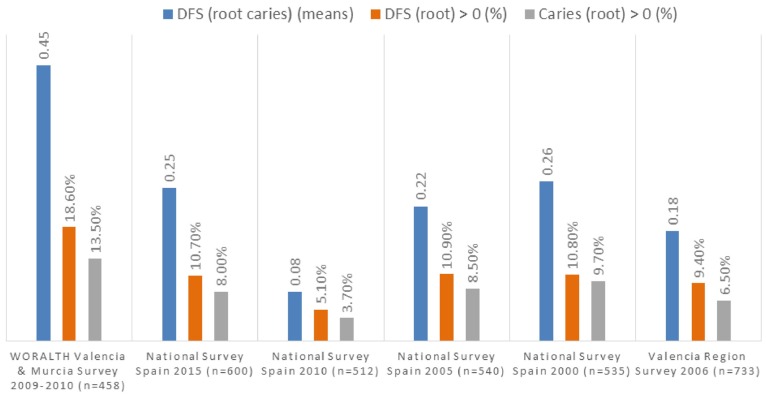
Comparison of root caries indices in adults of 35-44 years in Spain.

In this study, DFS (root) index was almost double than the observed in the national survey 2015 in Spain (0.45 vs. 0.25), and it was also higher than those from previous national surveys 2010 (0.08), 2005 (0.22) y 2000(0.26), and than the index observed in the study of the Valencia region (0.45 vs. 0.18); all studies showing a higher value of the D component (Decayed) vs. the F (Filled) component. In this study, observed root caries prevalence (18.6%) and active caries root prevalence (13.5%) were also considerably higher than those in the national surveys (Fig. 1). The explanation for the adverse results in this study, could be related to the sample distribution of working population, with a high percentage of subjects with manual occupations (60.5% manual vs. 39.5 non-manual) who, as it was previously described, showed worse root condition in every analyzed index; males also predominated in the sample (61.6% males vs. 38.4 females), and they showed higher prevalence of root caries and active root caries.

- Discussion on the influence of the studied variables on root caries

• Socio-demographic variables

The national epidemiological surveys in Spain, 2000 and 2005, in the cohort of young adults 35-44 years of age, did not find association between the root caries indices and the studied socio-demographic variables: gender, social level and geographical type of location (the samples had lower percentage of males than the sample in this study, 36.3% and 43.0%, respectively, vs. 61.6%).

In the 2010 national survey (42% males vs. 58% females) it was observed an association between the social level and geographical type and the two studied indices: root caries prevalence and caries index (active caries prevalence and DF components were not studied), that is, lower prevalence and index were observed in the higher social level and urban location.

In the 2015 national survey (40.8% males vs. 59.2% females) it was observed an association between social level and root caries prevalence, active root caries prevalence and DFS index. Nevertheless, it was not observed association between the other variables (i.e. gender and country of origin), neither was it observed for the 65-74 adults cohort.

For the senior adults cohort, 65-74 years, in the 2000 national survey (48.9% males) it was found an association between root caries prevalence, active root caries prevalence and DFS index, and gender (higher in males). In the 2005 national survey (51.5% males) an association between gender and active root caries prevalence was found. In the 2010 national survey (46.3% males) it was found an association between social level and root caries prevalence, and between gender, social level and geographical type of location and DFS index.

• Variables of frequency of toothbrushing and frequency of snacking/eating between meals 

In regard to the association between frequency of brushing and root caries prevalence in young adults (better root status in those who brushed more than once per day vs. those who brushed once per day or did not brush), other studies found higher root caries prevalence in both, young adults and senior adults, with high plaque levels (4,9,10), and in adults from 33 to 76 years of age (11), and other studies with lower brushing frequency in senior adults (1), and both in young and senior adults (12).

The habit of “snacking between meals” and root caries association is a logical and expected one, as it is associated with crown caries. The frequency of starches and sugar intake, and their permanence in the mouth environment, are considered more impor-tant than the sugar intake amount (13). The frequency of eating between meals (snacking) may have a statistically significant association with oral diseases (14). The consumption of soft drinks and sugary drinks has increased dramatically in developed countries, and nowadays, it could be an important factor for enamel erosion (15), making the cervical area more susceptible to these type of lesions. In other ranges of age, it was found an association between the frequency of eating and drinking with caries, for medium aged adults (from 45 to 64 years of age), but not for senior adults (over 65 years of age) (4).

• Alcohol and tobacco consumption variables

In regard to the alcohol consumption variable, in a study on Danish adults, the regression analysis showed a statistically significant association between the consumption of more than 15 alcoholic drinks per week with higher odds of root surface fillings, compared with no alcohol consumption (OR=1.7; *p*<0,001), especially in adults over 45 years of age (16).

On the other hand, it has been corroborated the influence of smoking >20 cigarettes per day, in active root caries prevalence, being tobacco a main factor associated with periodontal disease (6,17,18). There is a dose-response relationship between the number of cigarettes/day consumed and the number of years of smoking habit, and the severity of periodontal disease, where the effects on periodontal tissues seem to be more pronounced in males, than in females (19), which could be indicated by higher loss of attachment. A ten-year cross-sectional study on 65-85 adults found a positive relationship between root caries and the daily number of cigarettes consumed (20), as well as other studies have published the relationship of tobacco consumption and root caries in adults 21-89 years of age (16), and in adults 55-75 years (21). A prospective longitudinal study on Irish adults (mean age=69.1 years) suggested a correlation between root caries and poor plaque control, whereas they did not find a correlation with diet, smoking habit, alcohol consumption or education level (22).

Based on the results of the present study, we can conclude that in our sample of adults of 35-44 years, there is a higher prevalence of root caries in workers with manual occupations, foreigners, with primary studies, lower family income, who do not have the habit of brushing their teeth, who snack/eat between meals more than twice per day, and smokers of >10 cigarettes per day. Adult workers 35-44 years of age show worse root condition in regard to caries than general adult population of this age cohort. The frequency of toothbrushing and the frequency of snacking between meals are the variables that influence more in root caries, in this study.
